# Cell distribution after intracoronary bone marrow stem cell delivery in damaged and undamaged myocardium: implications for clinical trials

**DOI:** 10.1186/scrt4

**Published:** 2010-03-15

**Authors:** Virginie F Forest, Ashok M Tirouvanziam, Christian Perigaud, Sarah Fernandes, Marion S Fusellier, Jean-Claude Desfontis, Claire S Toquet, Marie-Françoise M Heymann, Dominique P Crochet, Patricia F Lemarchand

**Affiliations:** 1 INSERM UMR915, l'institut du thorax, IRT-Université de Nantes, 8 quai Moncousu, BP 70721, Nantes, F-44007 Cedex 1, France; 2 Université de Nantes, Faculté de Médecine, Institut Fédératif de Recherche Thérapeutique 26 (IFR26), 8 quai Moncousu, BP 70721, Nantes, F-44007 Cedex 1, France; 3 CHU Nantes, l'institut du thorax, Hôpital Nord Laënnec, Boulevard Jacques Monod, Nantes, F-44093 Cedex 1, France; 4 Animal Pathophysiology and Functional Pharmacology Unit (UPSP 5304), Ecole Nationale Vétérinaire de Nantes, Atlanpole La Chantrerie, Nantes, F-44307 Cedex 3, France; 5 CHU Nantes, Service d'Anatomo-pathologie, Hôpital Nord Laënnec, Boulevard Jacques Monod, Nantes, F-44093 Cedex 1, France; 6 INSERM U957, Laboratoire de Physiopathologie de Résorption Osseuse et Thérapie des Tumeurs Osseuses Primitives, Faculté de Médecine 1, rue Gaston Veil, Nantes, F-44035 Cedex 1, France

## Abstract

**Introduction:**

Early randomized clinical trials of autologous bone marrow cardiac stem cell therapy have reported contradictory results highlighting the need for a better evaluation of protocol designs. This study was designed to quantify and compare whole body and heart cell distribution after intracoronary or peripheral intravenous injection of autologous bone marrow mononuclear cells in a porcine acute myocardial infarction model with late reperfusion.

**Methods:**

Myocardial infarction was induced using balloon inflation in the left coronary artery in domestic pigs. At seven days post-myocardial infarction, 1 × 10(8) autologous bone marrow mononuclear cells were labeled with fluorescent marker and/or ^99m^Tc radiotracer, and delivered using intracoronary or peripheral intravenous injection (leg vein).

**Results:**

Scintigraphic analyses and Υ-emission radioactivity counting of harvested organs showed a significant cell fraction retained within the heart after intracoronary injection (6 ± 1.7% of injected radioactivity at 24 hours), whereas following peripheral intravenous cell injection, no cardiac homing was observed at 24 hours and cells were mainly detected within the lungs. Importantly, no difference was observed in the percentage of retained cells within the myocardium in the presence or absence of myocardial infarction. Histological evaluation did not show arterial occlusion in both animal groups and confirmed the presence of bone marrow mononuclear cells within the injected myocardium area.

**Conclusions:**

Intravenous bone marrow mononuclear cell injection was ineffective to target myocardium. Myocardial cell distribution following intracoronary injection did not depend on myocardial infarction presence, a factor that could be useful for cardiac cell therapy in patients with chronic heart failure of non-ischemic origin or with ischemic myocardium without myocardial infarction.

## Introduction

The possibility of tissue repair by autologous adult progenitor cells immediately captured the attention of clinicians confronted with the disabling, life-threatening circumstance of heart failure. During the last five years, more than a dozen clinical studies using bone marrow cells have been published, ranging from case reports to formal trials, deploying a range of differing cell-based therapies with the shared objective of improving cardiac repair [1].

Although most of these initial human trials suggest functional improvement, randomized clinical trials reported contradictory results [2,3] suggesting that the ideal protocol design still needs to be defined, with the help of large animal models [4]. Several issues have been identified by the European task force in translational research in the heart [5], including the type of cells to be used, the route of delivery, the number of cells and the volume to be given.

Our aim was, with an experimental design of acute myocardial infarction (MI) and autologous cardiac cell therapy that closely match clinical trials [2,3] to analyze and quantify whole body and heart distribution of injected autologous bone marrow-mononuclear cells (BM-MNCs). We also evaluated the optimal route of delivery (intracoronary *versus *peripheral intravenous injection).

## Materials and methods

### Animal procedures

Animal procedures were approved by the Institutional Committeefor Use and Care of Laboratory Animals at the Veterinary School ofNantes and conform to the *Guide for the Care and Use of Laboratory Animals *(NIH Publication No.85-23, revised 1996). Forty-six domestic pigs (35 to 40 kg) were included in the study. For MI induction and cell injection, anesthesia was induced by intramuscular injection of ketamine (10 mg/kg)/xylazine (2 mg/kg) and maintained with mechanical tracheal ventilation using inhaled isoflurane (2%). All animals received subcutaneously morphine (0.2 mg/kg) prior and after all procedures.

### Experimental myocardial infarction

We used the MI experimental model initially described by Suzuki *et al *[6], with slight modifications. Five days prior MI induction, animals received β-blocker carazolol (20 μg/kg) twice per day. Propranolol (0.05 mg/kg) was injected intravenously just before MI induction procedure to prevent arrhythmia. After the placement of a 6F sheath in the femoral artery, an extra-back up 6F guiding catheter (Boston Scientific Scimed, Inc, Boston, USA) was placed under fluoroscopy into the left main coronary artery. A 3 mm over the wire balloon catheter was placed either into the left anterior descending coronary artery distal to the first diagonal branch or into the left circumflex. After balloon inflation a coronary thrombus was induced by injections below the balloon of thrombin (900 UI, Sigma Aldrich Corporation, Saint Quentin Fallavier, France) and fibrinogen (5 mg, Sigma). The balloon was deflated two to three minutes after injections. Coronary occlusion was checked by angiogram, and MI was confirmed by ECG alteration and by plasma creatine kinase elevation 24 hours after MI induction (Reflotron, Roche Diagnostics, Meylan, France).

### Preparation and cryopreservation of autologous bone marrow mononuclear cells (BM-MNCs)

Five days after MI, a total of 100 mL of bone marrow was aspirated into heparin-treated Bone Marrow Aspiration/Intraosseous Infusion needle (15 Ga, 10 mm, Cardinal Health France 205 S.A.S, Châteaubriand, France) from the iliac crests and shoulder blades under general anaesthesia. Five points of puncture were performed at each site of 5 mL each. The duration of the procedure was 15 to 20 minutes. BM-MNCs were isolated using Ficoll gradient (Eurobio Les Ulis, France), and a total of 244 × 10^6 ^± 33 × 10^6 ^BM-MNCs was obtained.

BM-MNCs were cryopreserved in foetal calf serum 10% DMSO. Cryo-preservation for two days avoided two general anaesthesias the same day in seriously disabled animals and decreased the number of red blood cells to be injected.

### Dual fluorescence and radioactive labelling

Seven days after MI induction, BM-MNCs were quickly thawed and labeled immediately before injection using a green intracellular fluorescent dye, Carboxy Fluorescein diacetate Succinimidyl Ester (CFSE, Molecular Probes Invitrogen, Cergy Pontoise, France) [7].

^99m^Technetium (^99m^Tc) labeling was performed after CFSE labeling, using the Isolink™ kit according to the manufacturer's instructions (Mallinckrodt Medical B.V., Petten, the Netherlands). CFSE + BM-MNCs were incubated with Isolink™/^99m^Tc for 40 minutes at 37°C and pH = 6. Before injection, BM-MNCs were centrifugated and resuspended in 10 mL NaCl to obtain a final solution containing 300-400 MBq ^99m^Tc-CFSE + BM-MNCs. Less than 5% cell mortality was observed in all experiments using trypan blue exclusion method after thawing, after radiotracer and fluorescence labeling, and immediately before injection.

^99m^Tc cell leakage was evaluated before injection and after four hours incubation at 37°C *in vitro*. Every hour, supernatant was collected. ^99m^Tc activities were quantified on both supernatant and BM-MNC pellet using a gamma counter. Radio-labeling efficacy, defined as the percentage of ^99m^Tc cell activity in the final cell solution, was 98 ± 5% (n = 3) after four hours and was stable during the four hour experiment, suggesting no ^99m^Tc cell leakage.

### Autologous BM-MNC injection

At Day 7 after MI, a second catheterization was performed. A 3 mm over the wire balloon catheter was advanced to the site of MI induction. An angiogram was performed before completing the procedure. In the absence of coronary reperfusion, a recanalization was performed with the guide wire before its distal placement. BM-MNCs were injected less than 30 minutes after preparation. The balloon was inflated at low pressure (two bars) and 3.3 mL of labeled BM-MNCs were injected through the lumen. The balloon was deflated two to three minutes later. The same procedure was repeated three times with a two minute interval between procedures. A total of 10^8 ^BM-MNCs were injected in 10 mL of NaCl at a final concentration of 10^7 ^BM-MNCs/mL.

For intravenous delivery, 10^8 ^BM-MNCs were injected via the superficial vein of the left front leg, using a bolus injection of 15 seconds.

### Nuclear imaging and gamma counting

Planar scintigraphic images of ^99m^Tc-BM-MNC body distribution were obtained 1 hour and 24 hours after tracerinjection, using a gamma camera fitted with a low energy all purpose high resolution parallel hole collimator (DS7, Sopha Medical, GEMS, Buc, les Yvelines, France). The energy window was centred at 140 keV ± 20%. Using a dedicated image processing computer, a static image was recorded into a 64 × 64 matrix for 35 seconds. The camera was calibrated using a standard source measured with a gamma counter before each acquisition to convert image counts to activity in MBq. For each experiment a calibrated dose of technetium was used to quantify activity decay after 24 hours. Anesthetized animals were positioned in supine positions, at a constant distance of 30 cm from the collimator, and images were recorded in thoracic and abdominal projections. On each image, a region of interest was drawn around each organ to quantify counts within the drawn area. Organ activity in each region of interest was expressed as the percentage of the total initial injected activity (measured as the difference between the ^99m^Tc-BM-MNCs syringe content before and after injection) with correction for ^99m^Tc physical decay.

Animals were sacrificed, their organs were excised and imaged with the gamma camera, and activity was measured in weighted samples of each organ and used to quantify organ gamma counting radioactivity (ACAD activimeter, Lemer Pax, Nantes, France). All quantitative analyses were performed by two investigators blinded to animal treatment group.

### Pathologic examination and immunohistology

Animals were sacrificed 1 hour or 24 hours after BM-MNC injection. Heart samples were cryopreserved and sliced into contiguous transverse 5 μm thick cryosections or were embedded in paraffin and stained with Hematoxylin-Eosin-Safran (HES). Immunolabeling was performed to detect VonWillebrand Factor antibody (DakoCytomation SA. France, Trappes France). Cell nuclei were labeled using propidium iodide. Histology analyses were performed by two pathologists blinded to animal treatment group.

### Statistical analysis

Data were expressed as mean ± SD. The Kruskal-Wallis test was used for comparison of independent samples and Spearman's test for correlation coefficient. A value of *P *< 0.05 was considered statistically significant.

## Results

### Procedural results

**Figure 1:**
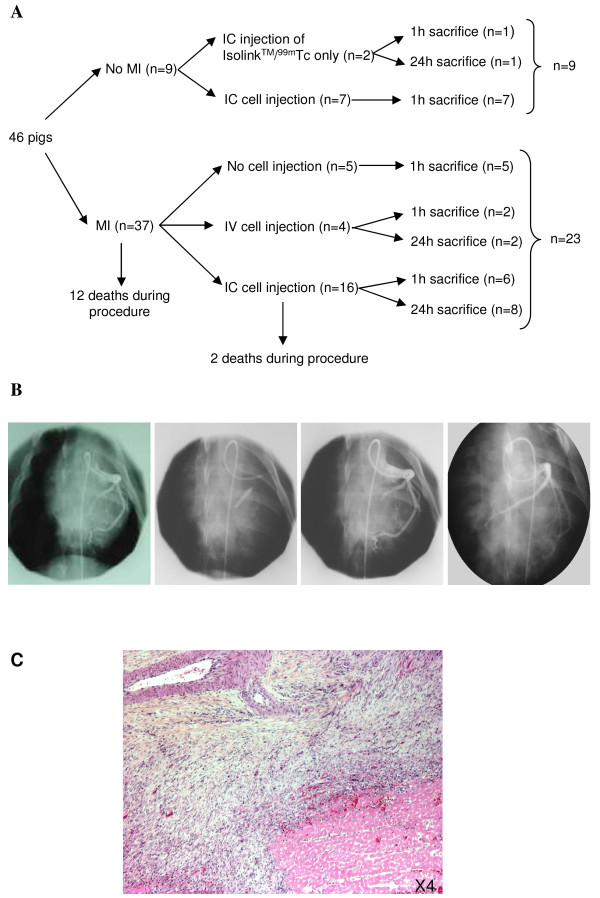
**Pig model of acute myocardial infarction**. **A**: Experimental design and animal groups. MI = myocardial infarction; IC = intracoronary delivery; IV = peripheral intravenous delivery. **B**: Induction of myocardial infarction. Coronary angiography, before, during, immediately after and seven days after occlusion of left anterior descending artery and fibrinogen/thrombin injections. **C**: HES staining of myocardial tissue seven days after MI induction, characterized by a central necrotic zone and an inflammatory response.

Myocardial infarction was induced in 37/46 pigs (Figure 1A). MI induction was complicated by death related to ventricular fibrillation during the procedure in eight cases (21.6%), and immediately after at the wake up time in four other cases (10.8%). Total death rate was 32% during and immediately after MI induction, probably due to extended MI [8].

The 25 surviving animals were randomly assigned to three different groups: five were used as controls (MI without BM-MNC injection), 16 received intracoronary BM-MNCs, four received intravenous BM-MNCs. Two deaths were observed during balloon inflation for BM-MNC intracoronary injection (one from a ventricular fibrillation and one from a second MI, probably related to the injection site location being within a non-infarcted area). In the 9/47 animals without MI induction, seven received intracoronary BM-MNC injection and two were used as radioactivity controls (intracoronary injection of Isolink™/^99m^Tc only).

### Myocardial infarction model

MI was induced by complete obstruction of the left coronary artery using balloon inflation and injection of thrombin and fibrinogen (n = 23; left anterior descending artery: n = 15; left circumflex artery: n = 8) (Figure 1B). Transient ST-segment elevation and isolated ventricular extrasystoles occurred during each coronary balloon occlusion, but abnormal ECG profile (ST-segment elevation) persisted only after injections of thrombin and fibrinogen. Plasma creatine kinase levels increased 24 hours after coronary occlusion (1,222 ± 604 UI/l before coronary occlusion, and 4060 ± 1961 UI/l at 24 hours, *P *< 0.01), confirming MI induction. Macroscopic examination of the heart performed seven to eight days after coronary occlusion showed extensive transmural antero-inferior MI encompassing 41.25 ± 5% of left anterior ventricle area (n = 23), with a thinning of the injured wall. Statistical analysis did not show any difference in MI size between animal groups, nor between animals with left anterior descending artery or left circumflex coronary occlusion, nor between animals with or without spontaneous reperfusion at the time of cell injection (not shown). Histopathologic examination confirmed MI, with associated inflammatory reaction and loss of cardiomyocyte nuclei (Figure 1C). Coronary angiography at seven days after MI showed spontaneous reperfusion in 18/23 pigs. Histological analyses showed an adherent residual thrombus within the coronary artery without total occlusion of the vessel lumen. Coronary occlusion in the five out of 23 remaining pigs required reperfusion before BM-MNC injection. Neither in pigs with spontaneous reperfusion nor in those with mechanical reperfusion, angiographic control showed abnomalities of blood flow distribution when compared to basal angiographic data obtained before MI induction.

### Biodistribution and ^99m^Tc-BM-MNC cardiac engraftment after MI and intracoronary injection

**Figure 2:**
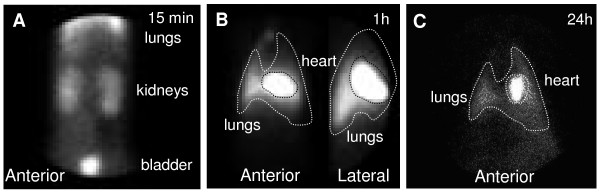
**^99m^Tc-BM-MNC biodistribution in animals with myocardial infarction after intracoronary injection**. **A-B**: abdomen (A) and thorax (B: anterior and lateral) scintigraphic images at 15 minutes and 1 hour. **C**: thorax scintigraphic image at 24 hours in the same animal.

Scintigraphic images were used to track ^99m^Tc-BM-MNCs after intracoronary injection. Radioactivity in the kidneys and the bladder due to free ^99m^Tc clearance, was observed 15 minutes after injection (Figure 2A). At one hour after injection, whole pig body images revealed intense regional accumulation of radioactivity in the heart (Figure 2B). At 24 hours a persistence of heart and lung radioactivity was observed, with a decrease in signal intensity (Figure 2C).

**Figure 3:**
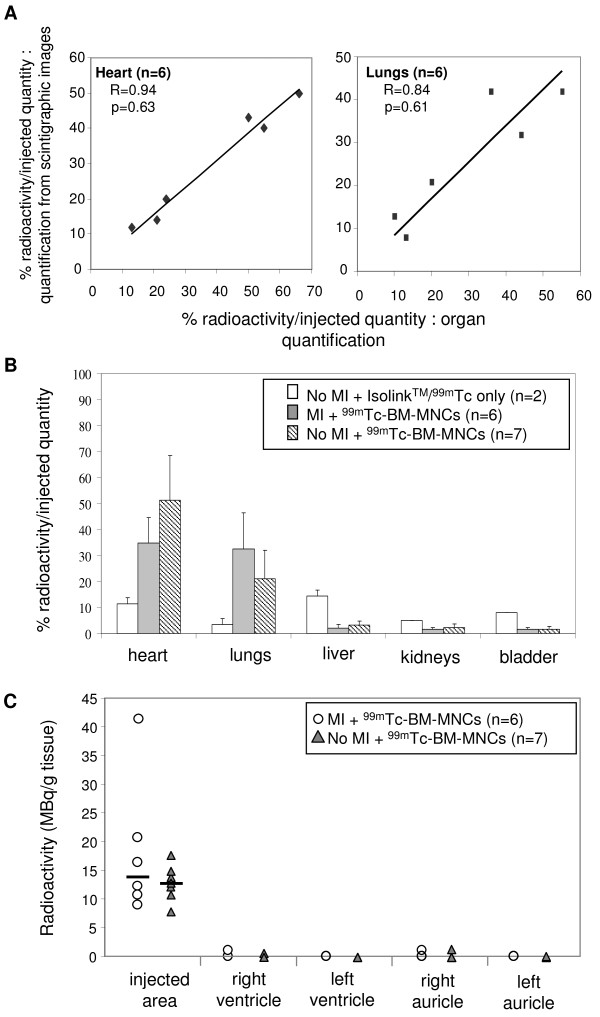
**^99m^Tc-BM-MNC biodistribution after intracoronary injection in animals with or without myocardial infarction**. The percentage of radioactivity in organs was calculated by dividing organ radioactivity with the total injected quantity after radioactive decay correction, one hour after injection. **A**: Comparison between two radioactivity quantification methods in the same animal: values from measured radioactivity in isolated organs (x-axis), and values from regions of interest drawn in the scintigraphic whole body acquisition images (y-axis). **B**: Radioactivity quantification in animals with (MI) or without (no MI) myocardial infarction, after intracoronary injection of Isolink™/^99m^Tc only or ^99m^Tc-BM-MNCs. Bars indicate mean and SD (*P *> 0.05, not significant). **C**: Individual quantities of radioactivity in pigs with or without MI (*P *> 0.05, not significant). Radioactivity was quantified in 1 g myocardium inside the infarcted area, in the left and right ventricles distant from infarcted area, and in the left and right auricles.

**Figure 4:**
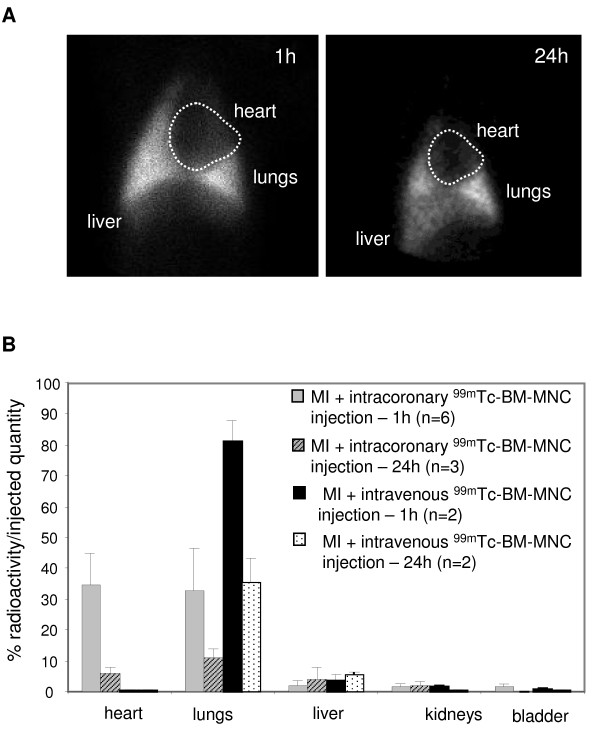
**^99m^Tc-BM-MNC biodistribution according to delivery route**. **A**: Scintigraphic acquisition images, 1 hour and 24 hours after peripheral intravenous ^99m^Tc-BM-MNC injection in the same pig with myocardial infarction. **B**: Radioactivity quantification, calculated by dividing organ radioactivity with the total injected quantity after radioactive decay correction. Bars indicate mean and SD.

Radioactivity was expressed as the fraction of measured radioactivity, decay-corrected and divided by the injected radioactivity amount. In the first set of experiments, radioactivity was quantified one hour after ^99m^Tc-BM-MNC intracoronary injection both in regions of interest drawn on the planar images and in isolated organs (Figure 3A). There was no significant difference between both quantification methods (n = 6, R = 0.94 for heart, and R = 0.84 for lungs). Therefore results from either method were used in following experiments. As controls, two pigs without MI but with intracoronary injection of Isolink™/^99m^Tc only were used to quantify free Isolink™/^99m^Tc fixation in each organ. At one hour radioactivity rate was 11.5 ± 2.1% in the heart, 3.5 ± 2.1% in the lungs and 14.5 ± 2.1% in the liver (Figure 3B), and at 24 hours radioactivity rate was null (not shown). Consistent with the *in vivo *scintigraphic images (Figure 2A), after ^99m^Tc-BM-MNC intracoronary injection high levels of radioactivity were measured in the heart (34.8 ± 9.9% of injected radioactivity) and in the lungs (32.6 ± 13.9%) (n = 6, Figure 3B). Small quantities of radioactivity were observed in other organs (2 ± 1.4% in the liver, 1.7 ± 0.6% in the kidneys). Radioactivity was also quantified in the infarcted site and in other cardiac tissues (right ventricle, healthy left ventricle and auricles, Figure 3C). We observed an accumulation of radioactivity in the infarcted area, Almost no radioactivity was detected remote from injection site (right ventricle, healthy left ventricle, auricles). 24 hours after BM-MNC injection, radioactivity persisted with lower intensity in the heart (6 ± 1.7%) and in the lungs (11 ± 2.6%) (n = 3, Figure 4B).

Importantly, there was no correlation between the infarction size and the heart radioactivity rate at one hour and 24 hours (not shown).

### Myocardial infarction and BM-MNC cardiac engraftment

To investigate the role of MI on BM-MNC homing and engraftment in the heart, ^99m^Tc-BM-MNC were intracoronary injected in pigs with MI and in pigs without MI. In both animal groups, ECG alteration (ST-segment elevation) was observed during intracoronary BM-MNC injection. ST-segment elevation stopped immediately after the procedure, suggesting that no micro-infarction was induced by injection itself favouring cardiac homing. Importantly, no significant difference in heart and lung BM-MNC distribution was observed between both groups, with a high radioactivity ratio in the heart (34.8 ± 9.9% in MI group *versus *51.2 ± 17.2% in no MI group, Figure 3B).

### Biodistribution of BM-MNCs and delivery route

The efficacy of intracoronary administration *versus *systemic intravenous administration was evaluated in pigs with MI. After intravenous cell delivery at 1 hour and 24 hours, radioactivity was detected mainly in the lungs both on scintigraphic images (n = 4, Figure 4A) and by radioactivity quantification, in marked contrast with intracoronary injection (Figure 4B). After intravenous injection, cardiac radioactivity was almost null (0.16 ± 0.23%) as compared to pigs after intracoronary injection (34.8 ± 9.9%). After 24 hours, no cardiac radioactivity was observed in intravenously injected pigs, suggesting an absence of cardiac homing at 24 hours.

### Histological analyses

**Figure 5:**
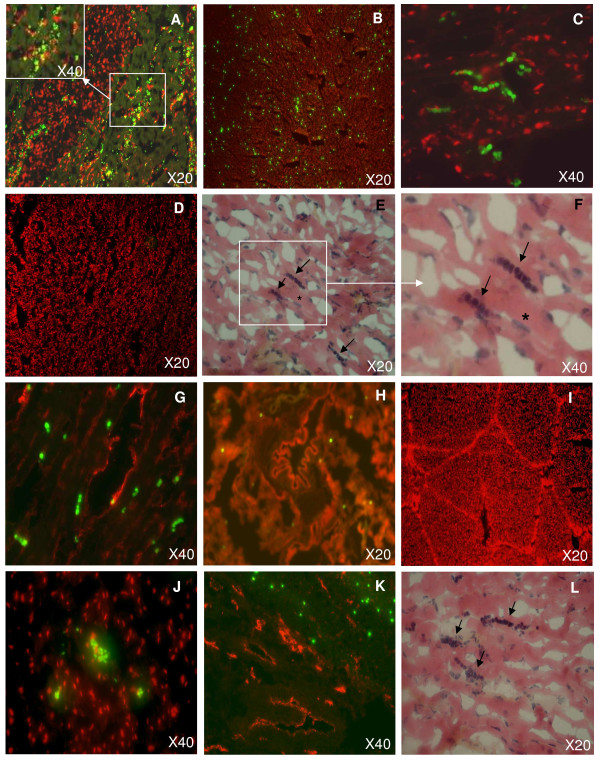
**Immunohistologic analyses**. Representative organ sections, after intracoronary injection of BM-MNCs labeled prior to injection with CFSE (green fluorescence). Cell nuclei were labeled with propidium iodide (red fluorescence). **A**: Fluorescent artefacts within the necrotic area of infarcted tissue. **B-C**: Myocardium sections of infarcted tissue, in absence of necrotic area, one hour after CSFE+ BM-MNC intracoronary injection. **D**: Myocardium section of a control animal (with myocardial infarction, without BM-MNC injection). **E-F**: The following section of C was stained with HES. Engrafted BM-MNCs within the infarcted zone appeared as round and dense blue points (arrow), in contrast to the elongated and blue pale cardiomyocyte nuclei (star). **G**: Myocardium section stained for endothelial cells and capillaries using anti-VonWillebrand antibody (red fluorescence). **H-I**: Lung (H) and liver (I) section. Cell nuclei labeled with propidium iodide (red fluorescence). **J**: Myocardium section of infarcted tissue, 24 hours after CFSE+ BM-MNC intracoronary injection. **K-L**: Myocardium section of an animal without myocardial infarction, one hour after CFSE+ BM-MNC intracoronary injection, using VonWillebrand antibody (K) or HES coloration (L).

For histological analyses, BM-MNCs were labeled with a fluorescent marker (CFSE) prior to injection and animals were sacrificed one hour or 24 hours after injection. As noticed by others [9] microscopic fluorescent artefacts were observed in the myocardium of control animals with MI and without BM-MNC injection, characterized by fluorescent leukocytes and autofluorescence within the infarcted tissue. However, these artefacts could be distinguished from BM-MNCs by their small size and their limited location within necrotic areas [10] (Figure 5A). BM-MNCs in non necrotic areas were detected in each animal that received CFSE+ BM-MNCs (Figure 5B-C), and were not observed in any control animals (with MI and without BM-MNC injection, Figure 5D). CFSE+ BM-MNCs were also observed in heart tissue 24 hours after intracoronary injection in each animal that received CFSE+ BM-MNCs (Figure 5J). The presence of BM-MNCs was confirmed in HES histological analyses (Figure 5E-F). BM-MNCs appeared as clusters of mononuclear cells, characterized by round and dense blue nuclei, in contrast with elongated and blue pale cardiomyocyte nuclei. These round and dense blue nuclei were not observed in control animals in HES histologic analyses (not shown). BM-MNCs were located in majority in the peri-infarcted myocardium and only a few BM-MNCs were detected in infarcted myocardium. No BM-MNCs were observed remote from the injection site (auricle, right ventricle, non infarcted myocardium).

Tissue sections were stained for endothelial cells and capillaries using anti-VonWillebrand antibody (Figure 5G). Neither thrombus nor arterial occlusion was observed in both groups. BM-MNCs were incorporated within myocardial tissue and were not observed within the lumen neither into wall blood vessel, and no cells were observed with double staining CFSE+ and endothelial marker. Double labeling for macrophages using anti-MAC-1 antibody was negative (not shown), suggesting that transplanted BM-MNCs had not been phagocyted.

We also investigated extracardiac BM-MNC biodistribution by histological analysis. CFSE+ BM-MNCs were disseminated in whole lungs (Figure 5H). Sections of other vital organs (liver, spleen, kidneys and thymus) demonstrated no significant cell location, only cell debris (Figure 5I).

Importantly, the presence of BM-MNCs was confirmed one hour (Figure 5K-L) and 24 hours (not shown) after intracoronary injection in every pig without MI and again, neither thrombus nor arterial occlusion was observed. These data were in accordance with analyses of scintigraphic data and radioactive organ quantification (Figure 3B).

The absence of cardiac homing after BM-MNC intravenous injection at 24 hours as observed on scintigraphic images was also confirmed by histological analyses, showing an absence of fluorescence in myocardium inside and outside MI area (not shown). In contrast, CFSE+ BM-MNCs were numerous and dispersed in whole lungs when injected intravenously (not shown).

## Discussion

In a porcine model of acute MI with late reperfusion and autologous cardiac cell therapy, we quantified biodistribution using a radiolabeling technique. Most of the BM-MNCs delivered by the intracoronary route were distributed within the heart and lungs. The presence of MI did not modify BM-MNC cardiac homing and engraftment after intracoronary injection. Finally, systemic intravenous BM-MNC injection promoted lung homing rather than cardiac engraftment.

The first prerequisite for cell therapy success is the engraftment and thus, homing of transplanted cells to the target area. Intracoronary delivery offers the advantages of a non-surgical method that can be performed percutaneously during angioplasty for acute MI. In the present study we showed that intracoronary BM-MNC injection can allow cell engraftment into myocardium tissue, whereas peripheral systemic intravenous administration was not efficient at 24 hours. This suggests that the cardiac niche effect favoured by MI was not sufficient to attract intravenously injected BM-MNCs within 24 hours, thus limiting the efficacy of systemic intravenous injection. Chemoattractant factors secreted by the infarcted heart might be too diluted in the body of large animals like pigs to attract BM-MNCs. If this is the case, similar results may be expected in humans. Another hypothesis is that the ability to trap intravenously injected BM-MNCs within the heart is limited, since only 4% to 5% of cardiac output is dedicated to supplying the coronary arteries [11]). Finally, cardiac engraftment following systemic intravenous BM-MNC delivery might be limited by lung entrapment prior to coronary artery access. Numerous studies have been performed in rodent [12] and large animal models [13,14], using intravenous injection of mesenchymal stem cells, showing mesenchymal stem cell engraftment after 24 hours. Several hypotheses can be discussed to explain these discrepancies with our results. First, our protocol closely matched clinical trial protocols, in which a seven-day delay between acute MI and cell injection seems to be the best time for efficacy following intracoronary injection [3,15]. However, this time may be too long after intravenous cell injection, in regards to local inflammatory response and niche effect. In most studies mesenchymal stem cells were injected 1 to 72 hours after myocardial infarction [12-14]. Second, BM-MNCs were labeled with ^99m^Technetium, a radioelement that has a short half life and may not have been detected at 24 hours. However, we did not observe any fluorescent cell within the heart 24 hours after intravenous injection, corroborating our radioactivity data. Finally, mesenchymal stem cells may have a better capability for cardiac homing than BM-MNCs, that contain a very small stem cell number. Interestingly, a recent study comparing intra-aortic, intravenous, and intramyocardial delivery of mesenchymal stem cells in rats observed 5% cell survival at 48 hours after intravenous delivery, mostly in the lungs [15].

The radioactive labeling using Isolink™ kit did not discriminate cell type, and was not species nor cell specific. In a pilot study we evaluated ^99m^Tc-hexa-methyl-propylen-amine-oxime (HMPAO) labeling, often used in humans. However, ^99m^Tc-HMPAO labeling of BM-MNCs was totally unsuccessful (not shown). We then switched to the radioactive linker Isolink™, which allowed us to inject a large quantity of ^99m^Tc-labeled cells, significantly higher than in other studies [16,17]. Although, similar to previous studies [11,18,19], the viability of Isolink™/^99m^Tc-labeled BM-MNCs was not altered, the adverse effects of labeling on the migratory and functional abilities of BM-MNCs cannot be entirely excluded. The majority of cardiac BM-MNC homing studies were performed in rodents, and only a few studies established the fraction of transplanted cells retained within the myocardium using direct radioactive labeling of the cells. In our study, radioactivity quantification in each organ showed 34.8 ± 9.9% total radioactivity in the heart with an accumulation within the injection site, one hour after intracoronary BM-MNC injection, and 6.0 ± 1.7% at 24 hours. Importantly, histology results were in accordance with scintigraphic imaging data confirming the presence of numerous BM-MNCs at one hour and 24 hours after intracoronary injection. As in our study, Hou *et al *[8], using a pig model of reperfused MI with intracoronary cell delivery and radioactive cell quantification, observed a largely right-sided distribution of BM-MNC. However, only 2.6 ± 0.3% of BM-MNCs were detected in the heart, with some BM-MNCs being distributed to the right ventricle. Importantly, the experimental model in this study was xenogeneic, with human BM-MNCs being injected into pigs. Human cells may lack the correct membrane receptors to home into the pig myocardium or may undergo acute lysis due to a xenogeneic reaction. In a recent study, using a similar model of reperfused MI in pigs with autologous BM-MNC transplantation [20] 6.5% of autologous transplanted BM-MNCs were detected in the heart four days after injection, a result close to ours at 24 hours. Finally, cardiac homing cell rate at one hour might be overestimated by including a possible leakage of radioactive label from the cells, although this appears unlikely as no radioactive cell leakage was observed *in vitro *in the next four hours following radioactive labeling.

Although phase I-II clinical trials have been completed using coronary delivery of BM cells, only one study used coronary delivery of ^99m^Tc-labeled BM-MNCs (in a single patient), and observed *intense *cardiac cell engraftment [17]. The study of Hofmann *et al *[11] showed that in five patients with MI, only 1.3 to 2.6% of ^18^F-FDG-labeled BM-MNCs were detected in the infarcted myocardium one hour after intracoronary BM-MNC injection. However, the number of injected BM-MNCs was 30-fold higher than in our study, so the absolute number of retained BM-MNCs within the heart was 39 to 78 × 10^6 ^BM-MNCs, a number close to ours (34.8 ± 9.9 × 10^6 ^retained BM-MNCs). Intracoronary injection of a very high cell number may saturate the binding sites for cardiac cell homing, raising questions about the ideal number of cells to be injected.

After intracoronary injection, we observed a similar biodistribution of BM-MNCs in animals with or without MI, despite the large size of the infarcted area (45% of the left ventricle). Several studies have shown that acute myocardial infarction is followed by an acute local inflammatory reaction involving upregulation of chemokines receptors and adhesion molecules, thereby facilitating adhesion and infiltration of cells involved in tissue repair, including stem cells [21-23]. The dynamic capability of BM-MNCs to migrate and the *niche *effect are central in regenerative medicine [21-23]. In our model, to carry out intracoronary injection, blood flow was stopped three times for at least two minutes to prevent backflow and prolong contact time between BM-MNCs and myocardium. When BM-MNCs were injected without prior MI induction, each maneuver for intracoronary cell injection resulted in ST-segment elevation, as already described for repeated angioplasty balloon inflations [24,25]. Although we did not evaluate the intensity of the ischemia induced by balloon inflation, this suggests that the injection technique created local downstream ischemic and preconditioning effects [25], thus rendering the local microenvironment more receptive to cell homing [24]. A recent study in a pig model of myocardial infarction and intracoronary injection of autologous BM cells, balloon occlusion was found ineffective to increase cell homing [24]. In another study in a similar model, single-bolus delivery was as effective as three balloon-occlusion deliveries [26]. Importantly, both studies were performed only in pigs with myocardial infarction and not in healthy pigs, suggesting that the cardiac niche effect favoured by MI was sufficient to attract injected BM-MNCs without any further effect of balloon occlusion. The fact that the presence and the size of MI did not influence cell engraftment could be useful for cardiac cell therapy in patients with chronic heart failure of non-ischemic origin or with ischemic myocardium without myocardial infarction. Two clinical trials have been published with patients who have had a myocardial infarction at least three months before BM-MNC coronary injection [1,27,28]. A clinical benefit was observed in both studies, suggesting that BM-MNCs homed to the myocardium despite the absence of acute myocardial infarction. The hypothesis of cardiac homing in the absence of acute myocardial infarction was recently confirmed in two Phase I cardiac cell therapy clinical trials on patients with chronic ischemic cardiomyopathy and receiving an intracoronary injection of radiolabeled CD133+ or CD34+ cells [16,29].

## Conclusions

Myocardial cell distribution following intracoronary injection did not depend on MI presence. Given these results, further efforts to analyze the mechanisms of adhesion and transmigration through the vascular wall will be rewarding.

## Abbreviations

^99m^Tc: ^99m^Technetium; BM-MNC(s): bone marrow-mononuclear cells; CFSE: carboxy fluorescein diacetate succinimidyl ester; DMSO: dimethyl sulfoxide; ECG: electrocardiogram; HES: hematoxylin-eosin-safran; HMPAO: hexa-methyl-propylen-amine-oxime; MBq: Mega Becquerel; MI: myocardial infarction; SD: standard deviation.

## Competing interests

The authors declare that they have no competing interests.

## Authors' contributions

VF carried out the cell therapy product preparation and drafted the manuscript. AT implemented the myocardium infarction animal model and carried out myocardial infarction inductions. CP implemented the cardiac cell therapy animal model and carried out bone marrow harvests. SF participated in the design of the study and helped to draft the manuscript. MF performed the analysis and interpretation of scintigraphic data. JCD carried out the radioactive experiments. CT participated to the conception and design of histological experiments. MFH carried out the analyses and interpretation of histological data. DC participated in carrying out in vivo experiments and helped to draft the manuscript. PL conceived of the study, and participated in its design and coordination, and drafted the manuscript. All authors read and approved the final manuscript.
